# Common variable immunodeficiency, same name but different etiologies: unraveling heterogeneity through functional and genetic profiling

**DOI:** 10.3389/fimmu.2026.1918470

**Published:** 2026-10-28

**Authors:** Celia Nieto-López, Lucía Dueñas-Prieto, Hye Sun Kuehn, Juan D. Acevedo-Ávila, Luis A. Andara, María J. Díaz-Madroñero, Kima López-Aldabe, Juan José Fernández-Díaz, Estela Paz-Artal, Rebeca Pérez de Diego, Rebeca Rodríguez-Pena, Mario Framil, Sergio D. Rosenzweig, Oscar Cabrera-Marante, Cristina Serrano, Luis M. Allende

**Affiliations:** 1 Department of Immunology, Hospital Universitario 12 de Octubre, Madrid, Spain; 2 Instituto de Investigación Sanitaria Hospital 12 Octubre (imas12), Madrid, Spain; 3 School of Medicine, Complutense University of Madrid, Madrid, Spain; 4 Immunology Service, Department of Laboratory Medicine, Clinical Center, NIH, Bethesda, MD, United States; 5 Department of Internal Medicine, Hospital Universitario Galdakao-Usansolo, Usansolo, Spain; 6 Biobizkaia Health Research Institute (IIS Biobizkaia), Bizakaia, Spain; 7 Interdepartmental Group of Immunodeficiencies, Madrid, Spain; 8 Laboratory of Immunogenetics of Human Diseases, IdiPAZ Institute for Health Research, La Paz Hospital, Madrid, Spain; 9 CIBER de Enfermedades Respiratorias (CIBERES), Instituto de Salud Carlos III, Madrid, Spain; 10 Department of Immunology, Hospital Universitario La Paz, Madrid, Spain; 11 Department of Immunology, Centre Diagnostic Biomedic, Hospital Clinic de Barcelona, Barcelona, Spain; 12 Department of Immunology, Fundación Jiménez Díaz, Madrid, Spain

**Keywords:** BLK, CVID - common variable immunodeficiency, functional validation, IKZF1, immune dysregulation, inborn error immunity, LRBA, primary immunodeficiencies

## Abstract

**Introduction:**

Common variable immunodeficiency (CVID) represents a challenge for medical genomics, as it does not fully conform to a classical Mendelian inheritance model. Beyond the monogenic model, diverse molecular mechanisms, including polygenic contributions, somatic mutations, epigenetic modifications, and skewed X-chromosome inactivation, may play relevant roles in disease development, driving a broad clinical spectrum from classical antibody deficiency to complex immune dysregulation. Integrating clinical, immunological, functional, and genetic data is therefore essential to unravel this complexity and advance toward personalized precision medicine.

**Methods:**

We conducted an in-depth clinical, immunological, functional, and genetic characterization of four distinct selected cases (P1–P4) from a cohort of adult patients with CVID. Targeted next-generation sequencing (NGS), cytokine profiling, and specific *in vitro* functional assays were performed to validate B-cell maturation defects, intracellular signaling, and variant pathogenicity.

**Results:**

We identified novel and rare pathogenic variants in key B-cell pathways that converged on a common B-cell maturation defect but diverged into distinct phenotypes. Patient 1 (P1) presented a novel *IKZF1* variant that disrupted early B-cell ontogeny, whereas Patient 2 (P2) exhibited a rare compound heterozygous defect in *TNFRSF13B* (TACI) that affected peripheral B-cell activation, both manifesting a predominantly infectious phenotype. In contrast, Patients 3 and 4 presented with severe immune dysregulation. Patient 3 (P3) harbored a novel splice-site deficiency in *LRBA*, while Patient 4 (P4) exhibited concurrent heterozygous variants in *BLK* and *EBF1*, supporting a novel oligogenic "double-hit" hypothesis. Functional assays validated the deleterious impact of these variants across all cases.

**Discussion:**

Our findings not only expand the known phenotypic spectrum but also underscore the value of functional assays in validating novel genotypes. Ultimately, this comprehensive profiling provides essential insights to refine prognosis and advance toward tailored precision medicine in CVID.

## Introduction

Common variable immunodeficiency (CVID) is the most prevalent symptomatic inborn error of immunity (IEI) in humans, with an estimated prevalence of between 1 in 20,000 and 1 in 50,000 (1). CVID is a heterogeneous disease, both clinically and immunologically, whose complexity is mainly due to variable penetrance and expressivity. The clinical spectrum is broad; however, two main phenotypes can be distinguished, one predominantly characterized by recurrent infections, present in the majority of patients, and another with autoimmune and/or inflammatory manifestations, affecting approximately 25-50% of patients. These complications represent a significant diagnostic and therapeutic challenge, as they are associated with increased morbidity and mortality compared to the infection-only phenotype (2–5).

Diagnostic criteria for CVID generally include reduced serum levels of IgG and IgA (or IgM), poor response to protein/polysaccharide vaccines or isohemagglutinins, and/or decreased switched-memory B cells, increased susceptibility to infections and the exclusion of secondary causes of hypogammaglobulinemia or profound T cell deficiency (6–9). Historically, due to its late onset of clinical symptoms and phenotypic heterogeneity, CVID has been considered a polygenic disorder. However, advances in whole exome and genome sequencing (WES/WGS) have enabled the identification of monogenic defects in approximately 20-30% of patients (10–12). To date, more than 50 genes involved in B-lymphocyte development, survival and immune tolerance have been identified (6, 10, 13).

The causes of CVID are diverse and often unclear; thus, an understanding of the biological processes that drive these disorders is essential for the diagnosis and treatment of complex cases. The biological effects of monogenic CVID-associated gene defects can be summarized as follows: impaired B-cell development and survival (*BAFFR, IKZF1, IRF2BP2, CD27, NFKB2, POU2AF1, STAT1 GOF, TWEAK*), impaired class-switch recombination and somatic hypermutation (*BACH2, IL21, IL21R*), excessive lymphoproliferation (*CTLA4, LRBA, PIK3CD, PIK3R1, PIK3CG, PTEN, STAT3* GOF), impaired B-cell activation, receptor signaling and costimulation (*BLK, CD19, CD20, CD21, CD81, ICOS, NFKB1, PLCG2, TNFRSF13B*) and others (*ARHGEF1, ATP6AP1, KARS1, MOGS, PI4KA, RAC2, SEC61A1, SH3KBP1, TRNT1*) (13–15).

Beyond monogenic defects, other mechanisms have been proposed to contribute to CVID pathogenesis, including oligogenic inheritance, epigenetic alterations, skewed X-chromosome inactivation, and somatic mutations (16–21). In the absence of a clear disease-causing germline mutation, the etiology remains elusive and although B-cell defects are the hallmark of the disease, T-cell abnormalities, such as decreased regulatory T cells or disturbed cytokine secretion, also occur, further complicating the pathophysiological spectrum (4, 7, 14).

In this context, the study of emerging genes and rare variants acquires special relevance, as many patients still lack a definitive molecular diagnosis. CVID exemplifies the paradigm of precision medicine, requiring the integration of clinical, immunological and molecular data to guide diagnosis and treatment. In the present work, we employ several molecular strategies to dissect the pathophysiological landscape of CVID by the characterization of four representative cases from our cohort harboring variants in *IKZF1*, *TNFRSF13B, LRBA* and *BLK* (in synergy with *EBF1*). These genes represent interconnected functional nodes governing B-cell ontogeny, activation, and immune homeostasis. By providing functional validation, our findings demonstrate how distinct molecular disruptions converge on a shared clinical phenotype, thereby expanding the genotypic spectrum of CVID and providing tools for its diagnosis.

## Materials and methods

### Study population and samples

This study includes a cohort of 80 adult patients diagnosed with CVID following the European Society for Immunodeficiencies (ESID) criteria. Peripheral blood samples were obtained from the four selected patients and healthy controls. Peripheral blood mononuclear cells (PBMCs) were isolated by density gradient centrifugation using Ficoll-Paque (Cytiva) for subsequent functional and molecular assays.

### Immunological evaluation

The immunophenotyping was performed on whole peripheral blood samples to assess conventional T, B and natural killer (NK) cell compartments. The T-cell compartment was evaluated based on the expression of CD45RA and CCR7 in CD4^+^ and CD8^+^ T cells. The B-cell compartment was assessed using IgD, IgM, CD27, CD38 and CD21 expression on CD19^+^ B cells. Reference values for immunophenotypic populations were obtained from Kverneland et al. (22). Cell acquisition was conducted using AQUIOS and DxFLEX flow cytometers (Beckman Coulter, Brea, CA, USA) and data were analyzed with Kaluza software version 2.1 (Beckman Coulter).

Serum total immunoglobulin concentrations (IgG, IgA and IgM) and IgG subclasses (IgG1, IgG2, IgG3 and IgG4) were determined by nephelometry using a Beckman Coulter analyzer according to the manufacturer’s instructions.

Specific antibody responses to protein and polysaccharide vaccines (including tetanus toxoid and *Streptococcus pneumoniae*) were evaluated by quantifying antigen-specific IgG titers using commercial ELISA kits (Binding Site, Birmingham, UK).

### Multiplex cytokine quantification

Plasma cytokine levels of IL-2R, IL-10, MIG (CXCL9), and IL-18 were quantified using a ProcartaPlex Human Custom High-Sensitivity Panel (Thermo Fisher Scientific, Waltham, MA, USA) on a Luminex 100 instrument (Luminex Corporation, Austin, TX, USA) according to the manufacturer’s protocol. Normal ranges were defined using a cohort of 12 age- and sex-mathced healthy adults controls.

### Genetic analysis

Genomic DNA was extracted from EDTA-anticoagulated peripheral blood using the QIAamp DNA Mini Kit (Qiagen, Hilden, Germany). DNA concentration was quantified using the Qubit dsDNA HS Assay Kit (Thermo Fisher Scientific).

For molecular germline screening, next-generation sequencing (NGS) was performed using a custom targeted panel of 476 genes associated with IEIs (SureDesign; Agilent Technologies, Santa Clara, CA, USA). Sequencing was carried out on the MiSeq platform (Illumina, San Diego, CA, USA), generating 75 bp paired-end reads with a mean depth of 100X and >98% coverage. Data processing, alignment (GRCh37/hg19) and variant calling were performed using Alissa Reporter and Interpret software (Agilent Technologies). Variant pathogenicity was assessed according to the updated guidelines of the American College of Medical Genetics and Genomics (ACMG) (23), considering allele frequency, *in silico* predictors (SIFT, PolyPhen-2, CADD), inheritance pattern probabilities (DOMINO) (24), immunological and functional data, familial segregation, and clinical phenotype. Candidate variants were visually inspected using the Integrative Genomics Viewer (IGV) (25) and validated by Sanger sequencing.

Furthermore, copy number variant (CNV) analysis was performed from targeted NGS data using Alissa Interpret software. A threshold of ≥ 5 consecutive targets was established to call CNVs were evaluated and classified following established recommendations for clinical practice.

### Gene expression assays

Total RNA was isolated from PBMCs using the ReliaPrep™ RNA Cell Miniprep System (Promega, Madison, WI, USA) and reverse-transcribed using the High-Capacity cDNA Reverse Transcription Kit (Thermo Fisher Scientific). Quantitative PCR (qPCR) was performed using TaqMan Fast Universal PCR Master Mix (Thermo Fisher Scientific) and gene-specific probes for *TNFRSF13B* (Hs00963364_m1), *BLK* (Hs01017458_m1), *EBF1* (Hs01092694_m1), *PAX5* (Hs05040337_s1), *CD79A* (Hs00998119_m1), and *CD79B* (Hs00236881_m1), with *GAPDH* (Hs02786624_g1) as the endogenous control. Relative expression was calculated using the comparative CT (2^-ΔΔCT^) method.

### Functional assays

For the B-cell receptor (BCR) phosphorylation assay, PBMCs were resuspended at 5 × 10^6^ cells/mL in RPMI-1640 medium supplemented with 2% fetal bovine serum (FBS) and stimulated 5 minutes at 37 °C with anti-human IgM F(ab’)_2_ fragments (20 µg/mL). Intracellular staining for phospho-Syk-PE (pY352), phospho-PLCγ2-PE (pY759), and phospho-ERK1/2-PE (pY204) (BD Biosciences, San Jose, CA, USA) was performed after fixation and permeabilization using the eBioscience™ Foxp3/Transcription Factor Staining Buffer Set (Thermo Fisher Scientific).

Surface TACI expression was measured by Mean Fluorescence Intensity (MFI) using anti-CD267-PE (Miltenyi Biotec, Bergisch Gladbach, Germany) on CD19^+^CD27^+^ gated B cells.

The functional impact of the *IKZF1* variant was evaluated using an ITGA5 luciferase reporter assay. Briefly, HEK293T cells were co-transfected with the wild-type (WT) or mutant *IKZF1* expression vector alongside the pEZX-PG04-ITGA5 promoter reporter plasmid (Genecopoeia, Cat. HPRM69441-PG04). Luciferase activity was measured using the Secrete-Pair Dual Luminescence Assay Kit (Genecopoeia) according to the manufacturer’s instructions on a GloMax^®^ Discover System GM3000 plate reader (Promega). *Gaussia* luciferase activity was normalized to secreted alkaline phosphatase activity to determine the transcriptional potency of the mutant protein. Immunofluorescence and electrophoretic mobility shift assays (EMSA) were performed as previously described (26).

To determine the functional relevance of the *LRBA* variant, a CTLA-4-dependent transendocytosis assay was performed. PBMCs from the patient and a healthy control were co-cultured for 24h with Chinese hamster ovary (CHO) cells expressing human CD80-GFP or CHO-WT cells as a negative control. Co-cultures were maintained in RPMI-1640 supplemented with 10% FBS under three conditions: (i) unstimulated, (ii) stimulated with anti-CD3/CD28 and IL-2 (6000 U/mL), or (iii) IL-2 alone. Following incubation, cells were stained for CD4-PB, CD25-PC5.5, FoxP3-APC and CTLA-4-PE using Treg Phenotyping Kit (Miltenyi Biotec). Samples were acquired on a BD FACSCanto II cytometer and analyzed using Kaluza software (Beckman Coulter). Transendocytosis efficiency was quantified as the percentage of CD4^+^CD25^+^FoxP3^+^CTLA-4^+^ T cells positive for CD80-GFP, and the MFI of GFP was used to assess total CD80 uptake. Additionally, LRBA protein expression was evaluated on CD45^+^ gated cells. Expression was assessed under basal (unstimulated) conditions and following cell activation which was confirmed by the upregulation of CD69. Protein levels were quantified by flow cytometry and expressed as MFI.

### Statistical analysis

Statistical analyses were performed using GraphPad Prism version 6.0 (GraphPad Software, USA). Data are presented as mean ± standard deviation (SD). For pairwise comparisons, continuous variables were analyzed using either the parametric two-tailed Student’s *t*-test or the non-parametric Mann-Whitney U test, as appropriate. For multiple gene transcript evaluation across groups, multiple unpaired t-test were applied with false discovery rate (FDR) correction using Benjamini-Hochberg approach. *P*-values less than 0.05 were considered statistically significant (* *p* < 0.05, ** *p* < 0.01, *** *p* < 0.001).

## Results

### Clinical and immunological characterization of the CVID cohort

At our center, 80 adult patients have been diagnosed with CVID according to the ESID criteria over the past 20 years (27, 28). Targeted NGS identified likely pathogenic or pathogenic variants in 15 of these 80 patients, achieving a diagnostic yield of 18.75%. The distribution of affected genes in our adult cohort reflects the genetic heterogeneity of CVID: *CTLA4* (n=3), *NFKB1* (n=2), *PIK3R1* (n=2), *TNFRSF13B* (n=2), *IKZF1* (n=1), *LRBA* (n=1), *BLK* (n=1), *MAGT1* (n=1), *LIG4* (n=1) and *STAT3* gain-of-function (n=1).

Although all identified variants were considered clinically relevant, four cases were selected for in-depth functional and phenotypic characterization based on their diverse defects affecting the B-cell lineage and development (**Figure 1**). These cases illustrate the broad clinical spectrum of the cohort, from classical antibody deficiency to complex immune dysregulation. Specifically, Patient 1 (P1) carries an *IKZF1* mutation that impairs early B-cell ontogeny, Patient 2 (P2) presents a *TNFRSF13B* defect affecting peripheral B-cell activation, Patient 3 (P3) suffers from an *LRBA* deficiency driving immune dysregulation and lymphoproliferation, and Patient 4 (P4) exhibits an oligogenic combination of *BLK* and *EBF1* mutations disrupting intracellular signaling.

**Figure 1:**
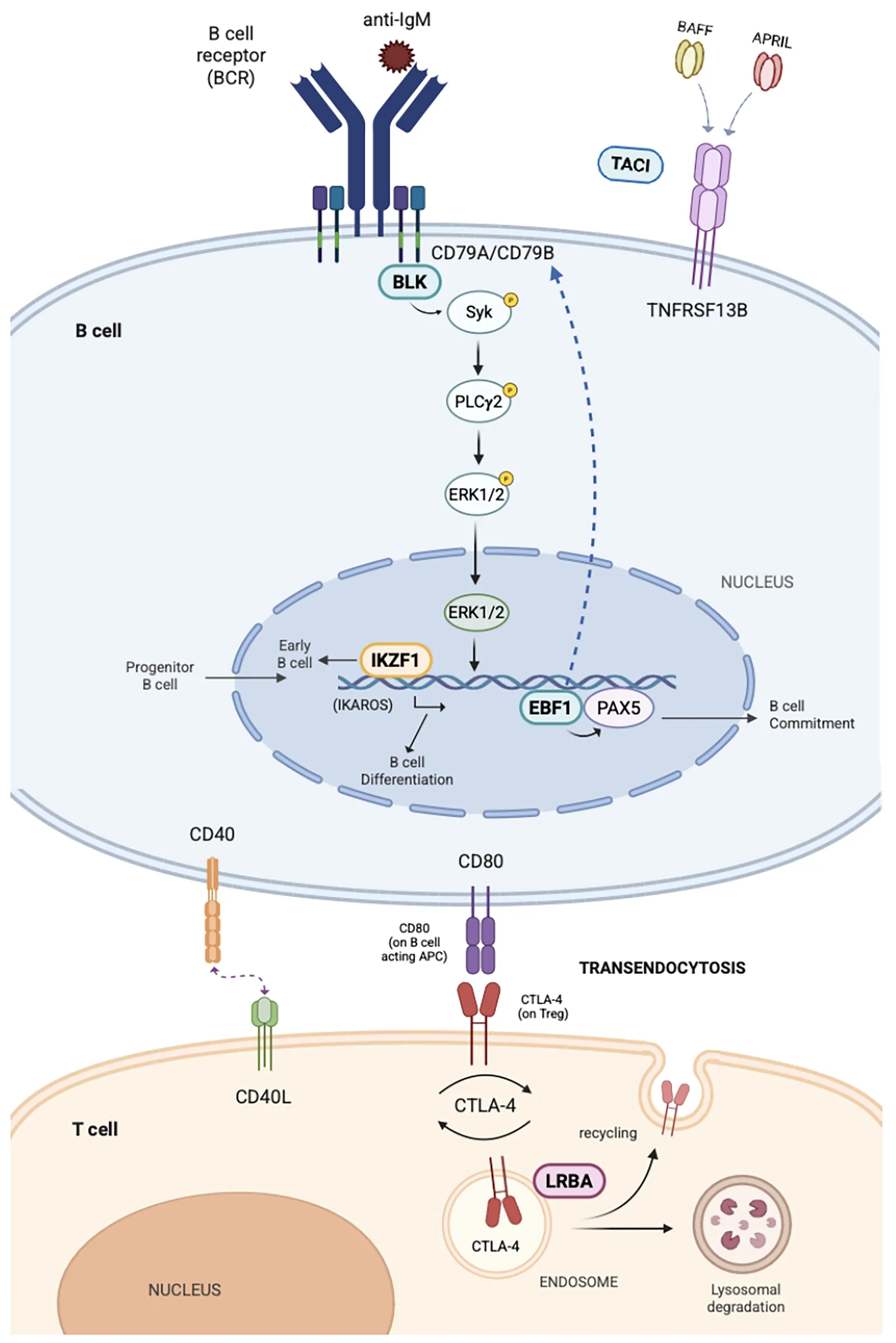
Integrated mechanistic model of B-cell and Treg cells pathways in CVID. Simplified representation of the molecular checkpoints and subcellular localizations of the proteins investigated in this study. (Top) B-cell surface receptor activation (TACI), BCR signalosome cascade (TACI, BLK, Syk, PLCγ2, ERK1/2), and nuclear transcriptional control (IKZF1, EBF1, PAX5, CD79A, CD79B) in early differentiation and lineage commitment. (Bottom) Immunological synapse and Treg homeostasis, highlighting LRBA-mediated CTLA-4 trafficking between the endosome and the cell surface (transendocytosis). Solid black arrows represent biochemical signaling or developmental flow. Blue dashed arrows represent signal transcriptional regulation. Purple dashed arrows represent binding interactions between cell surface molecules. Figure created with BioRender.com.

While all four patients shared the hallmark features of CVID, hypogammaglobulinemia and a severe reduction in switched-memory B cells (CD19^+^CD27^+^IgD^-^), their clinical and immunological profiles revealed two distinct disease phenotypes. (a) CVID with a predominantly infectious and intermediate phenotype: patients with IKZF1 (P1) and TACI (P2) deficiencies presented with classical CVID, where the primary clinical challenge was antibody deficiency and recurrent infections. P1 presented at age 45 with recurrent pneumonia and bronchiectasis. Her immunophenotype revealed a B-cell lymphopenia (CD19^+^: 5.9%; absolute count: 65 cells/μL) and a severe reduction of switched-memory B cells (3%). Beyond the B-cell defect, she exhibited an inverted CD4/CD8 ratio (0.8) due to a significant decrease in the absolute CD4^+^ count (380 cells/µL) and a profound reduction in naïve CD4^+^ T cells (7%). In contrast, P2 presented with a milder infectious burden alongside mild immune dysregulation features, including splenomegaly and immune thrombocytopenic purpura (ITP), with fluctuating IgG levels (600–750 mg/dL), reduction of switched-memory B cells (4%). Notably, P1 and P2 showed preserved appropriate vaccine responses to both polysaccharide and protein antigens (**Table 1**). (b) CVID with an immune dysregulation phenotype. Patients with LRBA (P3) and BLK/EBF1 (P4) deficiencies showed a complex clinical profile dominated by autoimmunity and lymphoproliferation. P3 presented with an early-onset syndrome characterized by severe multi-organ autoimmunity (nephritis, inflammatory brain lesions, and lymphocytic interstitial pneumonia), ITP, autoimmune cytopenia, lymphadenopathy, and splenomegaly. Immunologically, he displayed a profound dysregulation profile with an expansion of CD21^low^ B cells (14.6%), low naïve CD4^+^ and CD8^+^ T cells (8.15% and 12.36%, respectively), and T-cell activation, particularly in the CD8^+^ compartment (CD8^+^HLA-DR^+^: 58.72%). Further deep phenotyping revealed diminished regulatory T-cell (Treg) and B-cell (Breg) compartments, with low Treg (CD4^+^CD25^+^CD127^low^: 2.18% [normal range: 4.9-7.4%]), reduced Breg (CD19^+^CD24^hi^CD27^+^: 15.01% [normal range: 22.5-27.1%]), and nearly absent FoxP3^+^ expression (0.4% [normal range: 2.7-7.1%]). Similarly, P4 presented with ITP, evolving to Evans syndrome, interstitial lung disease (ILD), splenomegaly and systemic lymphadenopathy. Despite a normal total B-cell count (117 cells/µL), she showed a severe arrest in B-cell maturation, with a marked decrease in switched-memory B cells (0.7%). Additionally, the T-cell compartment exhibited both CD4^+^ (358 cells/μL) and CD8^+^ (100 cells/μL) lymphopenia, alongside a reduction in naïve CD4^+^ T cells (30.7%) and (**Table 1**).

**Table 1:** Immunophenotype, cytokines and humoral evaluation of CVID cases.

parameters	patient 1 (*ikzf1*)	patient 2 (*tnfrsf13b*)	patient 3 (*lrba*)	patient 4 (*blk/ebf1*)	reference values
Total lymphocytes	**1085**	1629	1724	**731**	1200-3000/µL
CD3^+^	**83** (906)	**65** (1064)	**85.85** (1512)	**65 (477)**	68-76% (850-2250/µL)
TCR alfa/beta	91	95	92.51	97	85-99%
TCR gamma/delta	**9**	5	**7**	2	2.9-5.5%
CD3^+^TCR alfa/beta^+^CD4^-^CD8^-^ (DNT)	0.2	0.9	1.23	3.7	0-3.5%
CD4^+^	**35 (380)**	**47** (769)	**40.33** (695)	**49 (358)**	54-69% (500-1450/µL)
CD4^+^CCR7^+^CD45RA^+^ (N)	**7**	58.1	**8.15**	**30.7**	39-53.7%
CD4^+^CCR7^+^CD45RA^-^ (CM)	**77**	**15.1**	**75.47**	41.4	30.7-40.4%
CD4^+^CCR7^-^CD45RA^-^ (EM)	15	25.8	15.85	26.5	7.2-15.3%
CD4^+^CCR7^-^CD45RA^+^ (TEMRA)	0.25	0.99	0.54	1.4	0.2-2.8%
CD8^+^	45 (485)	**16** (263)	39.43 (680)	**14 (100)**	25-39% (160-950/µL)
CD8^+^CCR7^+^CD45RA^+^ (N)	**21**	**71.5**	**12.36**	56.9	29.2-65.9%
CD8^+^CCR7^+^CD45RA^-^ (CM)	**20**	**1.6**	16.25	16.5	6.2-14.1%
CD8^+^CCR7^-^CD45RA^-^ (EM)	24	20.8	36.66	16	16.6-34.6%
CD8^+^CCR7^-^CD45RA^+^ (TEMRA)	35	6.1	34.72	10.6	5.9-38.1%
CD4/CD8	**0.8**	2.9	1.02	3.6	1.1-2.6
CD4^+^HLA-DR^+^	24	3.59	16.42	**26.84**	4.1-12.7%
CD8^+^HLA-DR^+^	65	4.59	**58.72**	42.34	9.1-44.6%
CD19^+^	**5.9 (65)**	31 (505)	**6.22** (112)	16 (117)	8-14% (100-500/µL)
CD19^+^CD27^+^	**12**	**24.9**	21.98	**11.2**	17.1-22.7%
CD19^+^IgD^+^CD27^-^ (N)	**86.3**	**74.1**	**71.76**	**86.7**	38.4-62.9%
CD19^+^IgD^+^CD27^+^ (MZ)	8.6	20.8	22.92	11.7	9.8-18.9%
CD19^+^IgD^-^CD27^+^ (SM)	**3**	**4.0**	**1.92**	**0.70**	11.6-17.9%
CD19^+^CD38^++^IgM^++^CD27^-^ (Transitional)	**7.4**	1.24	**0.04**	**6.12**	1-2.5%
CD19^+^CD21^low^	**1.2**	**1.20**	**14.58**	6.60	3.8-9.7%
CD56^+^CD16^+^CD3^-^ (NK)	8.6 (93)	3.1 (50)	4.48 (81)	16.1 (117)	4-20% (60-450/µL)
IgG	**509**	**603**	**663**	**402**	700–1600 mg/dL
IgA	**25**	**39**	**<1**	**<5**	40–230 mg/dL
IgM	**38**	**41**	110	**30**	70–400 mg/dL
IgG1	ND	387	715	490	280–800 mg/dL
IgG2	ND	183	**45**	**99**	115–570 mg/dL
IgG3	ND	98	241	70	24–125 mg/dL
IgG4	ND	**3.8**	**<0.4**	**<0.5**	5–125 mg/dL
Vaccine response	Adequate	Adequate	Impaired	Impaired	
IL-2R	1775	22	**29908**	**26116**	0–1900 pg/mL
IL-10	0	0	1	**7**	0–5 pg/mL
IL-18	27	28	**68**	**84**	0–30 pg/mL
MIG (CXCL9)	<0.75	<0.75	10	**285**	0–30 pg/mL

Altered values are shown in bold. DNT, double negative T-cell; N, naïve; CM, central memory; EM, effector memory; TEMRA, terminal effector memory re-expressing CD45RA; MZ, marginal zone; SM, switched-memory; ND: not determinable. Reference values were obtained from Kverneland et al. (22).

Finally, the plasma cytokine profile of the patients was determined to validate the stratification between the two clinical phenotypes. Cytokine quantification revealed a dichotomy that mirrored the clinical phenotypes. Patients with a predominantly infectious phenotype (P1 and P2) maintained low levels of both soluble IL-2 receptor (sIL-2R: 1,775 pg/mL and 22 pg/mL, respectively) and IL-18 (27 pg/mL and 28 pg/mL, respectively). In contrast, patients with the immune dysregulation phenotype (P3 and P4) presented a marked increase of the levels of both sIL-2R (29,908 pg/mL and 26,116 pg/mL, respectively) and IL-18 (68 pg/mL and 84 pg/mL, respectively), indicating a state of chronic activation related to the development of autoimmunity and inflammation in this subgroup (29). Furthermore, this inflammatory signature was particularly pronounced in P4, who also exhibited elevated levels of the chemokine MIG/CXCL9 (258 pg/mL) and the immunoregulatory cytokine IL-10 (7 pg/mL), which remained low or undetectable in the remaining patients (**Table 1**).

### Identification of germline mutations in the *IKZF1, TNFRSF13B, LRBA* and *BLK*/*EBF1* genes

A targeted NGS panel of 476 genes related to IEI was used to explore a monogenic cause of CVID. We identified four distinct genetic variants involving critical pathways of the B-cell development (**Table 2**).

**Table 2:** Genetic findings in selected CVID cases.

Patient ID	Gene	RefSeq	HGVS coding	HGVS protein	Consequence	Zygosity	VAF (%)	Inheritance	Classification	ACMG criteria
P1	*IKZF1*	NM_006060.4	c.524G>A	p.Cys175Tyr	Missense	Heterozygous	51	AD	LP	PS3 (Strong) PM1 (Moderate) PM2 (Supporting) PP3 (Supporting)
P2	*TNFRSF13B*	NM_012452.3	c.236A>G/ c.310T>C	p.Tyr79Cys/ p.Cys104Arg	Missense Missense	Compound heterozygous	48/ 53	AR/AD	LP/ LP	PS3 (Strong) PM3 (Moderate) PM2 (Supporting) PP3 (Supporting) PS3 (Strong) PM5 (Moderate) PP3 (Supporting)
P3	*LRBA*	NM_001364905.1	c.8468 + 1G>C	–	Splice donor	Homozygous	100	AR	P	PVS1 (Very strong) PS3 (Strong) PM2 (Supporting)
P4	*BLK*	NM_001715.3	c.403G>T	p.Glu135*	Stop gained	Heterozygous	38	AR/AD	LP	PVS1 (Strong) PS2 (Strong) PM2 (Supporting) PP3 (Supporting)
	*EBF1*	NM_001290360.2	c.1471G>A	p.Gly491Arg	Missense	Heterozygous	44	AD	LP	PS2 (Strong) PM2 (Supporting) PM2 (Supporting) PP2 (Supporting) PP3 (Supporting)

Variants are described using HGVS (Human Genome Variation Society) nomenclature. ACMG (American College of Medical Genetics and Genomics) criteria were applied for variant pathogenicity classification following current guidelines (Richards S et al., 2015). VAF, variant allele frequency; AD, autosomal dominant; AR, autosomal recessive; LP, likely pathogenic; P, pathogenic; PVS, pathogenic very strong; PS, pathogenic strong; PM, pathogenic moderate; PP, pathogenic supporting. Accession GenBank numbers: P1 (PZ577944), P2 (PZ577945), P3 (PZ577946), P4 (PZ577947 and PZ577948).

For Patient 1 (P1), a novel germline heterozygous missense variant was identified in the *IKZF1* gene (c.524G>A; p.Cys175Tyr). This variant is located in the third zinc-finger of the N-terminal DNA-binding domain, a region critical for the transcriptional regulation of lymphocyte differentiation. Segregation analysis confirmed the absence of the *IKZF1* variant in the patient’s mother and son. As the father was unavailable for testing, the variant is presumed to have arisen *de novo*. Although other pathogenic variants affecting this specific Cys175 residue have been recently described (26), the p.C175Y substitution identified in our patient represents a novel genetic variant, further expanding the mutational landscape of IKAROS.

Patient 2 (P2) was compound heterozygous for two previously reported variants in the *TNFRSF13B* gene, encoding TACI (Transmembrane Activator and CAML Interactor): c.236A>G (p.Tyr79Cys) and c.310T>C (p.Cys104Arg), inherited from the father and mother, respectively. Individually, both variants have been described as modifiers of CVID susceptibility rather than definitive monogenic causes (30). While each variant alone is probably insufficient to cause disease, their biallelic inheritance may contribute to the clinical phenotype. As expected, heterozygous relatives carrying only one of the variants were asymptomatic.

In Patient 3 (P3), a novel homozygous splice-site variant was identified in the *LRBA* gene (c.8468 + 1G>C). This mutation affects the highly conserved donor splice site of intron 56 and disrupts mRNA processing, leading to a premature stop codon and loss-of-function (LoF) of the LRBA protein, consistent with an autosomal recessive inheritance model. Notably, P3 was born to consanguineous parents (first cousins). Although segregation analysis could not be performed, the variant is presumed to be inherited from both parents.

Regarding Patient 4 (P4), a novel heterozygous nonsense variant in the *BLK* gene (c.403G>T; p.Glu135*) was identified, located in the SH3 domain. Furthermore, a second heterozygous missense variant was found in the *EBF1* gene (c.1471G>A; p.Gly491Arg). This *EBF1* variant was initially identified in a lymph node biopsy and subsequently confirmed in peripheral blood. To rule out a somatic origin, buccal mucosa cells were sequenced, confirming the germline nature of both variants. Segregation analysis confirmed the absence of both the *BLK* and *EBF1* variants in the patient’s mother and brother. As the father was unavailable for testing, both variants are presumed to have arisen *de novo*. The coexistence of these two defects suggests an oligogenic model affecting early B-cell commitment and BCR signaling.

Variant interpretation was performed using data from population databases (gnomAD, HGMD), and *in silico* prediction tools (SIFT, PolyPhen-2, CADD). All variants were validated by Sanger sequencing. According to ACMG guidelines (23), the *LRBA* variant was classified as pathogenic, while the *IKZF1, TNFRSF13B, BLK* and *EBF1* variants were categorized as likely pathogenic.

### Molecular and functional characterization of the *IKZF1, TNFRSF13B, LRBA* and *BLK/EBF1* variants

To confirm the pathogenic impact of the identified variants, we performed a series of functional assays tailored to evaluate the biological function of each affected gene, evaluating their effects from transcriptional regulation to cellular homeostasis (**Figures 1**, **2**).

**Figure 2:**
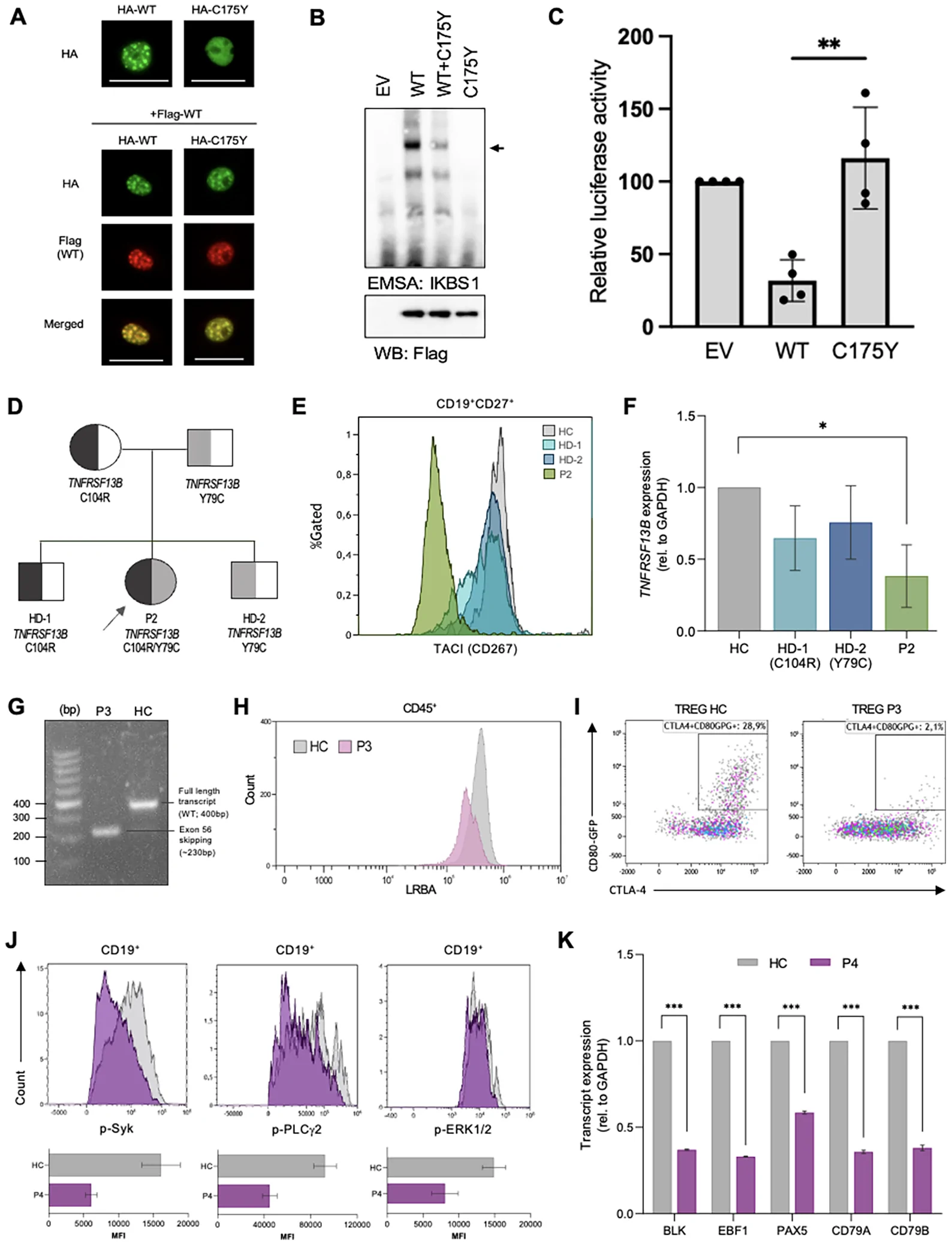
Functional characterization of CVID cases with *IKZF1*, *TNFRSF13B, LRBA* and *BLK/EBF1* variants. **(A)** Representative fluorescence microscopy image of NIH3T3 cells transfected with the HA-tagged WT or C175Y mutant constructs, either alone or co-transfected with Flag-tagged IKAROS WT. Scale bars indicate 25 μm. **(B)** Electrophoretic mobility shift assay (EMSA) using nuclear extracts from HEK293T cells transfected with the indicated plasmid. The arrow indicates IKAROS-containing complexes, likely representing dimers. **(C)** *ITGA5*-luciferase reporter activity in HEK293T cells overexpressing IKAROS WT or the mutant C175Y. Data represent the mean ± standard deviation from four independent experiments. Significance was determined by two-tailed Student t test (** *p* < 0.01). **(D)** Familial pedigree of P2, showing compound heterozygous inheritance of *TNFRSF13B* c.310T>C (p.C104R) and c.236A>G (p.Y79C) variants. Asymptomatic heterozygous carrier siblings are indicated as HD-1 and HD-2. The arrow identifies the proband (P2). **(E)** Surface expression of TACI (CD267) on memory B cells (CD19^+^CD27^+^) from P2, heterozygous carrier siblings (HD-1 and HD-2) and HC, assessed by flow cytometry. **(F)** *TNFRSF13B* transcript expression in P2, HD-1 and HD-2, determined by RT-qPCR and expressed relative to HC (performed on PBMCs, where the percentage of B cells was highly consistent across all samples). Data represent the mean ± standard deviation from four independent experiments. Significance was determined by Mann-Whitney U test (* *p* < 0.05). **(G)** RT-PCR analysis showing aberrant splicing (exon 56 skipping) of *LRBA* mRNA in P3 compared to HC. **(H)** LRBA protein expression determined by flow cytometry in CD45^+^ cells from P3 and HC. **(I)** CTLA-4 transendocytosis assay using CHO-CD80-GFP cells. Percentage of CD80-GFP capture by stimulated Tregs cells (CD4^+^CD25^+^FoxP3^+^CTLA-4^+^) from P3 compared to HC. **(J)** B-cell receptor (BCR) signaling in P4 and healthy control (HC) assessed by median fluorescence intensity (MFI) of phosphorylated downstream signaling molecules Syk, PLCG2 and ERK1/2 in CD19^+^ B cells after anti-IgM stimulation. **(K)** Transcript levels of *BLK, EBF1, PAX5, CD79A* and *CD79B* in P4, determined by RT-qPCR and expressed relative to HC. Data represent the mean ± standard deviation from three independent experiments. Significance was determined by multiple unpaired t-test using Benjamini-Hochberg FDR approach (*** *p* < 0.001). HC group is matched in sex and age.

We first evaluated the impact on IKZF1, a transcription factors essential for B-cell development. To characterize the *IKZF1* variant identified in P1, we generated a mutant construct to assess the functional activity of the IKAROS C175Y protein. EMSA assays revealed that the mutant completely failed to bind its target sequences. This loss of function was further explored by immunofluorescence: whereas wild-type (WT) IKAROS exhibited a punctate staining pattern consistent with pericentromeric heterochromatin (PC-HC) targeting, the C175Y mutant displayed a diffuse nuclear pattern, suggesting impaired pericentromeric localization. To mimic the patient’s heterozygous state, WT and mutant proteins were co-expressed. The absence of interference with WT localization suggested that the mutant does not exert a dominant-negative effect and likely behaves through haploinsufficiency. This was further supported by luciferase reporter assays, where the mutant failed to exert its physiological transcriptional suppressive function (**Figures 2A–C**).

Next, we examined the functional impact of a co-stimulatory surface receptor essential for B-cell activation. For P2 (TNFRSF13B), a compound heterozygous for the C104R and Y79C variants, we evaluated this defect by analyzing both mRNA and protein expression. RT-qPCR showed markedly reduced *TNFRSF13B* transcript levels in P2, while her heterozygous siblings, carrying only one of the variants (C104R or Y79C), exhibited intermediate mRNA levels. This transcriptional defect correlated closely with the surface expression of TACI (CD267) on memory B cells (CD19^+^CD27^+^), where P2 displayed a reduction in MFI compared to the intermediate levels in her siblings and normal expression in HC (MFI: 51,635 vs 137,744 vs 141,856 vs 176,794, respectively). This gene-dosage effect suggests that while single variants act as modifiers, their biallelic inheritance in P2 results in a critical loss of TACI function, which could directly contribute to the CVID phenotype (**Figures 2D–F**).

Regarding the variants affecting protein trafficking and receptor homeostasis, we evaluated the molecular consequences for P3 (*LRBA*). cDNA analysis by Sanger sequencing confirmed that the *LRBA* c.8468 + 1G>C mutation disrupts physiological splicing, leading to the skipping of exon 56. This molecular defect resulted in significantly reduced LRBA protein expression in lymphocytes, as measured by flow cytometry, with a more pronounced defect observed under unstimulated conditions (MFI 228,306 vs HC 372,664). To assess the impact on immune tolerance, we performed a CTLA-4 transendocytosis assay. Regulatory T cells (Tregs) from P3 exhibited a profound failure in capturing CD80-GFP (2.1% vs 28.9% in HC), accompanied by lower surface levels of CTLA-4 (MFI: 4095 vs 10886). These results confirm that the LRBA defect severely impairs the immune-regulatory capacity of Tregs (**Figures 2G–I**).

Finally, we investigated the cumulative effect of the identified double-hit defect disrupting both BCR signaling and its nuclear transcriptional control for P4 (*BLK/EBF1*). To evaluate the impact of the *BLK* nonsense variant, we quantified mRNA levels in PBMCs by RT-qPCR, which revealed a substantial reduction in transcripts, suggesting nonsense-mediated decay (NMD). To determine whether this reduction translated into signaling defects, we analyzed the B-cell receptor (BCR) cascade by assessing the phosphorylation of key downstream signaling molecules by flow cytometry. Upon anti-IgM stimulation, P4 showed a marked reduction in the phosphorylation of Syk, PLCγ2, and ERK1/2 in CD19^+^ cells compared to healthy control (HC), indicating impaired BCR signaling. Furthermore, to explore the impact on the *EBF1*-dependent transcriptional program, we analyzed the expression of *EBF1* and its downstream targets. RT-qPCR analysis demonstrated a generalized downregulation of *EBF1, PAX5, CD79A*, and *CD79B*. Although the percentage and absolute count of B cells were within normal ranges, P4 exhibited a profoundly distorted B-cell subset distribution. Given that this transcript analysis was performed on PBMCs, we acknowledge that this coordinate reduction in B-cell restricted genes may partly reflect this altered compartmental architecture alongside potential variant-driven transcriptional impairment. Overall, these combined signaling and expression defects align with previous reports of *BLK* deficiency in CVID (31) and highlight the critical role of the BLK/EBF1 axis in maintaining B-cell function (**Figures 2J, K**).

## Discussion

CVID is characterized by a highly heterogeneous genetic etiology, representing a major diagnostic and therapeutic challenge. Despite significant advances in NGS technologies, a considerable proportion of patients with a clinical diagnosis of CVID still lacks a definitive molecular diagnosis, particularly those carrying rare or non-classical variants whose pathogenicity is difficult to establish. In this study, the functional characterization of variants in *IKZF1*, *TNFRSF13B*, *LRBA*, and *BLK/EBF1* provides crucial insights into how these specific molecular defects lead to B-cell dysfunction and clinical penetrance in our cohort.

The study of Patient 1 (P1) focuses on IKAROS, encoded by *IKZF1*, a master transcription factor essential for early lymphocyte specification, late-stage B-cell differentiation, and the maintenance of central B-cell tolerance (32). Germline mutations in *IKZF1* are associated with primary immunodeficiency and can be classified into four different mechanisms of action depending on the protein expression and its functional defects: haploinsufficiency, dimerization defective, dominant-negative effect, and gain of function. These distinct molecular mechanisms are associated with diverse clinical outcomes, correlating with variable degrees of susceptibility to infectious diseases, autoimmune disorders, allergic diseases, and malignancies (33).

A subset of *IKZF1* mutations, particularly loss-of-function (LoF) variants resulting in haploinsufficiency, has been linked to CVID, which typically presents with recurrent infections, hypogammaglobulinemia, progressive loss of B cells, and autoimmune diseases (34, 35). In P1, we characterized a novel heterozygous missense variant (p.C175Y) located within the essential N-terminal zinc-finger DNA-binding domain (ZF3). Functional assays demonstrated that while the mutant C175Y protein preserves its nuclear localization, it exhibits a profound loss of DNA-binding capacity as assessed by EMSA, which directly correlates with a failure in transcriptional suppression, as evidenced by the *ITGA5*-luciferase reporter assay. Crucially, when co-transfected alongside *WT* IKAROS, the C175Y mutation failed to exert a dominant-negative effect, establishing that the underlying pathogenic mechanism in P1 is functional haploinsufficiency. This molecular behavior allows for a clear pathophysiological differentiation from other *IKZF1*-allelic variants. Unlike the dominant-negative *IKZF1* mutations described by Boutboul et al., which typically cause severe combined immunodeficiency affecting the T, B, and myeloid lineages (36), the haploinsufficiency in P1 results in a phenotype restricted to the B and T-cell compartment, with infections as the sole clinical manifestation. Furthermore, this loss-of-function defect contrasts with the gain-of-function variants characterized by Hoshino et al., which hyperactivate IKAROS and can alter late differentiation programs (37). Additionally, Stoddard et al. recently demonstrated that IKAROS actively regulates human T-cell phenotypes and post-thymic homeostasis, providing a crucial mechanistic link to the immune dysregulation and abnormal T-cell phenotype frequently observed in these patients (38). Ultimately, the quantitative transcriptional restriction caused by the functional p.C175Y haploinsufficiency appears to compromise both B-cell differentiation and peripheral T-cell homeostasis, driving the clinical manifestations observed in P1.

In contrast to classical monogenic disease-causing models, other variants identified in our cohort are considered genetic susceptibility factors, such as the *TNFRSF13B*/TACI variants in Patient 2 (P2). TACI represents one of the most frequently mutated genes in CVID, identified in approximately 8–10% of patients (39). However, its role as a monogenic disease driver remains highly controversial given that monoallelic *TNFRSF13B* variants have been reported in nearly 1% of the healthy population, supporting its classification as a susceptibility gene with incomplete penetrance where additional genetic or environmental factors may contribute to disease development (30, 40). In P2, we identified a compound heterozygous state for the well-known variants p.C104R and p.Y79C. Our data could illustrate a gene-dosage effect. While the asymptomatic heterozygous sibling carriers (HD-1 and HD-2) maintained preserved TACI surface expression and normal transcript levels, P2 exhibited a severe reduction in both mRNA and surface protein expression on memory B cells. These results are consistent with previous studies suggesting that certain *TNFRSF13B* missense variants, particularly in compound heterozygosity, affect receptor function or expression (39–42). Interestingly, P2 presented with a mild elevation of peripheral B lymphocytes. This finding aligns with both human and murine TACI-deficient models, which demonstrated that loss of the regulatory feedback exerted by TACI leads to B-lymphocyte activation and expansion, potentially driving clinical manifestations such as lymphadenopathy, splenomegaly, and, in some cases, autoimmunity (43, 44). Consequently, while monoallelic variants are relatively common and their pathogenicity remains debated, the biallelic inheritance in P2 has a molecular impact that destabilizes TACI expression, thereby disrupting immune homeostasis and converting a genetic susceptibility into overt clinical CVID.

Within the clinical spectrum of CVID, phenotypes characterized by immune dysregulation represent the most complex manifestations, which may be linked to defects in receptor recycling, as observed in Patient 3 (P3). P3 carries a novel homozygous splice-site variant in *LRBA* (c.8468 + 1G>C) resulting in complete exon 56 skipping. LRBA deficiency disrupts peripheral tolerance by compromising its role as a molecular chaperone that rescues CTLA-4 from lysosomal degradation within regulatory T cells (Tregs) (45). At the protein level, the exon skipping identified in P3 was associated with reduced basal LRBA expression in unstimulated lymphocytes, a finding consistent with quantitative defects described in the resting state of pathogenic variants (46),. To evaluate the functional impact, we performed a CTLA-4-dependent transendocytosis assay by flow cytometry using CHO cells expressing CD80-GFP (47). Tregs from P3 showed a marked inability to deplete and internalize CD80 from antigen-presenting cells compared to healthy controls. This impairment in transendocytosis provides an explanation for the patient’s clinical phenotype, characterized by early-onset multi-organ autoimmunity, systemic lymphadenopathy and splenomegaly. Functional LRBA deficiency depletes the surface availability of CTLA-4, preventing the competitive removal of co-stimulatory ligands (CD80/CD86). This regulatory failure correlates directly with the reduction of the patient’s Treg and an expansion of CD21^low^ B cells, a key cell population driven by persistent chronic stimulation and linked to systemic autoimmunity within the phenotypic spectrum of LRBA deficiency (48). Concurrently, this chronic lymphocyte-driven inflammatory environment is further reflected at the systemic level by the elevation of plasma sIL-2R and IL-18 observed in this patient (29, 49).

The most complex genetic profile in our cohort was observed in Patient 4 (P4), who carries a double heterozygous defect in *BLK* (p.Glu135*) and *EBF1* (p.Gly491Arg). *BLK* (B-lymphocyte tyrosine kinase) encodes a non-redundant tyrosine kinase of the Src family, crucial for early B-cell receptor (BCR) signal transduction (31), whereas *EBF1* (Early B-cell Factor 1) is a master transcription factor essential for B-cell lineage commitment and the maintenance of B-cell identity (50). In P4, the heterozygous *BLK* nonsense variant (p.Glu135*) resulted in a substantial loss of transcripts, suggesting nonsense-mediated decay (NMD). Crucially, this structural loss translated into a functional defect in the downstream BCR cascade, as evidenced by the decreased phosphorylation of Syk, PLCγ2, and ERK1/2 upon anti-IgM stimulation. In parallel, our exploration of the *EBF1* pathway revealed a transcriptional impairment, with a generalized downregulation of *EBF1* and its essential downstream targets *PAX5*, *CD79A*, and *CD79B*. Clinically, this molecular disruption correlates with a distinct plasma inflammatory profile in P4, characterized by concurrent elevations of sIL-2R, IL-18, MIG/CXCL9 and IL-10, a signature typical of immune dysregulation CVID phenotype associated with autoimmunity and lymphoproliferation (29, 41).

Although *in silico* algorithms predict these genes in *BLK* and *EBF1* to follow ambiguous AD/AR and AD inheritance patterns, respectively, and despite *BLK* variants having been described in rare cases of CVID (31), population genetics and animal models do not support a classical monogenic etiology for these individual variants. Querying public databases such as gnomAD reveals hundreds of healthy individuals carrying predicted loss-of-function (pLoF) mutations in *BLK*, and the gene displays a low probability of loss-of-function intolerance (pLI = 0), which contradicts the haploinsufficiency hypothesis. Furthermore, *Blk* knockout mice show no overt immune defects due to functional compensation by redundant Src kinases (51). Similarly, despite the high DOMINO dominance score for *EBF1* (24) and while its complete absence arrests early B-cell development, heterozygous mice exhibit mild hematopoietic phenotypes, with traits often restricted to non-immune tissues (52).

Given these constraints, neither the *BLK* variant nor the *EBF1* variant alone appears sufficient to cause full clinical CVID. Consequently, we propose that *BLK* and *EBF1* act as genetic susceptibility factors operating through an oligogenic “double-hit” model. The *EBF1*-mediated transcriptional downregulation of *CD79A* and *CD79B* reduces the physical co-receptor substrate available for docking, while the concomitant absence of BLK prevents the remaining complex from reaching the activation threshold required for memory B-cell differentiation. This molecular synergy outlines a novel oligogenic pathway where *BLK* acts as a potent disease modifier when combined with structural transcriptional impacts.

Notably, these four distinct genetic etiologies, which encompass defects in transcriptional factor (IKZF1), TNF-receptor family signaling (TNFRSF13B), vesicular trafficking and Treg function (LRBA), and a combined kinase/transcriptional factor axis (BLK/EBF1), all converge on a common cellular endpoint: the arrest of B-cell maturation and the marked reduction of switched-memory B cells (CD19^+^IgD^-^CD27^+^). However, the plasma cytokine profile demonstrates that this shared cellular block does not translate into a uniform clinical entity but rather reveals a dichotomy separating an infectious phenotype from a severe immune dysregulation phenotype characterized by elevated levels of sIL-2R, IL-18 and CXCL9/MIG. This dichotomy underscores that behind identical peripheral B-cell subset alterations lie distinct clinical phenotypes, ranging from predominantly humoral failure to severe immune dysregulation. Ultimately, unraveling these specific molecular drivers emphasizes that CVID frequently functions as a provisional, exclusion-based phenotypic diagnosis. The identification of a precise genetic etiology enables patient reclassification, transitioning from the predominantly antibody deficiency group to diseases of immune dysregulation or their specific molecular categories within the IUIS classification (13).

We acknowledge several limitations of our study. First, the cohort size is inherently limited to four characterized cases within a single-center design, which may restrict the generalizability of our observational cytokine dichotomy. Second, while long-term clinical follow-up was available for certain cases, prolonged prospective monitoring will be necessary to fully evaluate clinical penetrance over time. Regarding P4, while transcriptional and signaling defects support an oligogenic model involving BLK and EBF1, the EBF1 p.Gly491Arg variant was not functionally evaluated in an isolated, variant-specific assay, and the observed gene downregulation may partially reflect alterations in peripheral B-cell subsets. Finally, the proposed gene-dosage effect for TNFRSF13B in P2 is based on patient-level expression comparisons that still need to be formally validated in larger cohort designs.

In summary, our study highlights that the clinical landscape of CVID is determined by a broad spectrum of immune disorders, ranging from germline monogenic defects with variable penetrance to complex oligogenic interactions. While the *IKZF1* and *LRBA* variants in our cohort represent clear monogenic factors with distinct molecular mechanisms, the *TNFRSF13B* and *BLK*/*EBF1* variants underscore the need to assess gene dosage and multilocus synergy. Beyond this germline landscape, our findings reinforce the emerging paradigm that additional genetic mechanisms, specifically somatic mutations and epigenetic dysregulation, act as critical checkpoints in CVID pathophysiology, directly disrupting immune tolerance and driving clinical dysregulation (21, 53). Genetic testing is essential not only for establishing an accurate molecular diagnosis in complex or pediatric CVID cases, but also for identifying patients at high risk for complications, such as autoimmunity or malignancy (54). Finally, integrating germline, somatic and epigenetic data into precise functional and transcriptional networks enhance our understanding of the genetic architecture of these conditions, potentially facilitating future targeted immunomodulatory therapies and improved disease stratification.

## Data Availability

The datasets presented in this study can be found in online repositories. The names of the repository/repositories and accession number(s) can be found below: https://www.ncbi.nlm.nih.gov/genbank/, SUB16278686 https://www.ncbi.nlm.nih.gov/genbank/, SUB16279141 https://www.ncbi.nlm.nih.gov/genbank/, SUB16279210 https://www.ncbi.nlm.nih.gov/genbank/, SUB16279250 https://www.ncbi.nlm.nih.gov/genbank/, SUB16279415.
